# Learning lessons in sepsis from the children

**DOI:** 10.15252/msb.20188335

**Published:** 2018-05-17

**Authors:** Steven Timmermans, Claude Libert

**Affiliations:** ^1^ VIB Center for Inflammation Research Ghent Belgium; ^2^ Department Biomedical Molecular Biology Ghent University Ghent Belgium

**Keywords:** Network Biology, Pharmacology & Drug Discovery, Systems Medicine

## Abstract

Sepsis research has had relatively limited therapeutic success so far. In their recent study, Kobzik and colleagues (Joachim *et al*, 2018) identify novel drug‐sensitive pathways in sepsis, derived exclusively from patient data. Their strategy is based on the analysis of a naturally sepsis‐resistant population (pre‐puberty children) and on the implementation of a novel‐rich Pathway Drug Network, constructed from human gene expression data enriched in drug–pathway–gene clusters.

Sepsis is a dysregulated host response to infection that potentially leads to organ failure, blood pressure decline, and death. The yearly number of cases worldwide is estimated to be at least 20 million. With a mean lethal response of 25%, targeting sepsis is clearly a major unmet medical need.

Sepsis research is considered to be notoriously difficult. Over the past decades, huge investments have been made both by academic researchers and pharmaceutical companies, but no obvious candidate drugs have survived clinical trials and hit the market. Several companies have had major financial drawbacks due to these failed attempts, and therefore, the fear for investment in sepsis research is substantial. One can wonder why these immense efforts have not led to therapeutic success so far. While one can criticize the setup of many clinical trials (Opal *et al*, 2014), a major issue is the translation of pre‐clinical animal work to the real clinical situation. The use of animal models such as mice is well motivated, but the differences in response to sepsis between mice and men may be simply too big (Seok *et al*, 2013). Moreover, there are substantial basic physiological differences between mice and humans. For example, given the fact that sepsis involves a significant metabolic crisis, mice, which have a much larger surface/volume ratio than men, go into hypothermia, while humans do not. Also, the husbandry of mice in animal houses with specific pathogen‐free conditions may lead to very unnatural gut microbiota compositions, leading to immature deviant immune responses compared to the natural outside world (Beura *et al*, 2016; Wilmore *et al*, 2018).

Hence, research breakthroughs yielding new candidate drug targets, by directly analyzing human sepsis patient data, are important but remain rather underexplored. In their recent study, Kobzik and colleagues (Joachim *et al*, 2018) describe such a successful endeavor. The innovative aspect of their work is twofold (Fig 1). First, they focus on two patient groups that respond quite differently to clinical sepsis, namely pre‐puberty children that are known to be significantly less sensitive to sepsis‐induced lethality compared to the second group, post‐puberty adults. The reduced response to sepsis in pre‐puberty children is a well‐known phenomenon amongst doctors (Watson *et al*, 2003) and is in fact not limited to sepsis, but applies to many infectious diseases in general. Not much is known about the underlying mechanisms involved. The authors righteously consider this relative resistant pre‐puberty population as a sort of “experiment of nature” that the research community could interrogate. Secondly, Joachim *et al* (2018) employed a pathway‐level approach, rather than a common gene‐level analysis, and processed their patient transcriptomics results with a Pathway Drug Network (PDN). This PDN was newly constructed form drug–gene, disease–gene, drug–disease, and pathway–gene interaction sets, along with the expression data of more than 58,000 human publically available microarrays from the Gene Expression Omnibus (GEO) database.

**Figure 1:**
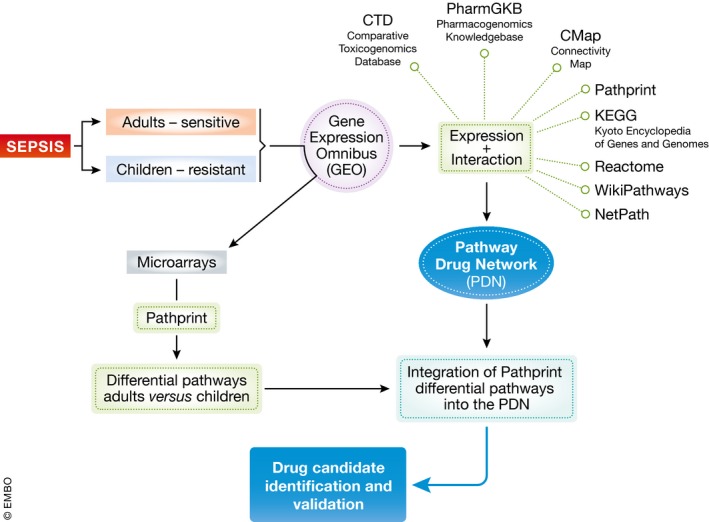
Resistance to sepsis in children links to candidate drugs via a rich Pathway Drug Network The workflow used in the study by Joachim *et al* (2018) involves two key aspects. First, the authors constructed the PDN, based on the Comparative Toxicogenomics Database (CTD), the Pharmacogenomics Knowledgebase (PharmGKB), the Connectivity Map (CMap), and Pathprint. Second, they used this PDN to compare differential pathways between children and adults undergoing sepsis, to identify candidate drugs, which were then validated in a mouse model.

Their unique PDN method was applied on pathways identified in microarray data obtained from 167 adult (18–91 years old, mean age 59) and 95 pre‐puberty sepsis patients (5–11 years old, mean age 8) (via Pathprint). The four main pathway clusters (up‐regulated in children, down‐regulated in adults; down‐regulated in children, up‐regulated in adults; up‐regulated in children, unchanged in adults and down‐regulated in children, unchanged in adults) were used to extract subnetworks from their PDN that correspond to the pathways that differ between the two patient groups. They were then able to obtain a ranking of potential drug candidates. They selected the top 10 candidate drugs that were associated with the pathways that differ between pre‐ and post‐puberty patient microarray profiles. Most of these 10 compounds had never been linked to sepsis before, and indeed, several conferred some protection in a mouse model of endotoxemia. Of course, it remains to be seen whether some of these drugs will lead to a breakthrough in sepsis management in human patients.

The innovative aspect of this approach is that the authors are able to predict pathways which are important in sepsis, as well as drugs associated with these pathways, exclusively based on sepsis gene expression data of blood samples of two groups of patients with a different disease response. There are no animal models involved. Although one may argue that the bulk of the data in the PDN network are derived from cell lines or from diseases with little or no association with sepsis, nevertheless, the drug to gene pathways to disease links are extremely rich and form the basis of the success of the study. Moreover, the application of the PDN network to sepsis is likely only one example of the spectrum of applications of this approach. If we consider this approach as a proof of principle, it will be interesting to see which other drug targets will be identified for sepsis from follow‐up studies, using larger patient groups and more sepsis subgroups. Indeed, one may expect other results from (and other drugs for), say a pneumonia compared to a meningitis or a bladder sepsis patient.

Clearly, the construction of the PDN database could serve as inspiration for future approaches. For example, in modern intensive care units, hundreds of measurements are obtained from sepsis patients each day of hospitalization (blood pressure, temperature, blood chemistry, organ function readouts, etc.). This collection of data, as well as RNA‐seq data, when integrated with PDN‐like networks in future analyses, could likely enhance the approach described by Joachim *et al* (2018).
